# Gut Microbiome Response to Sucralose and Its Potential Role in Inducing Liver Inflammation in Mice

**DOI:** 10.3389/fphys.2017.00487

**Published:** 2017-07-24

**Authors:** Xiaoming Bian, Liang Chi, Bei Gao, Pengcheng Tu, Hongyu Ru, Kun Lu

**Affiliations:** ^1^Department of Environmental Health Science, University of Georgia Athens, GA, United States; ^2^Department of Environmental Sciences and Engineering, University of North Carolina at Chapel Hill Chapel Hill, NC, United States; ^3^Department of Population Health and Pathobiology, North Carolina State University Raleigh, NC, United States

**Keywords:** artificial sweetener, sucralose, gut microbiota, metabolomics, inflammation

## Abstract

Sucralose is the most widely used artificial sweetener, and its health effects have been highly debated over the years. In particular, previous studies have shown that sucralose consumption can alter the gut microbiota. The gut microbiome plays a key role in processes related to host health, such as food digestion and fermentation, immune cell development, and enteric nervous system regulation. Inflammation is one of the most common effects associated with gut microbiome dysbiosis, which has been linked to a series of human diseases, such as diabetes and obesity. The aim of this study was to investigate the structural and functional effects of sucralose on the gut microbiota and associated inflammation in the host. In this study, C57BL/6 male mice received sucralose in their drinking water for 6 months. The difference in gut microbiota composition and metabolites between control and sucralose-treated mice was determined using 16S rRNA gene sequencing, functional gene enrichment analysis and metabolomics. Inflammatory gene expression in tissues was analyzed by RT-PCR. Alterations in bacterial genera showed that sucralose affects the gut microbiota and its developmental dynamics. Enrichment of bacterial pro-inflammatory genes and disruption in fecal metabolites suggest that 6-month sucralose consumption at the human acceptable daily intake (ADI) may increase the risk of developing tissue inflammation by disrupting the gut microbiota, which is supported by elevated pro-inflammatory gene expression in the liver of sucralose-treated mice. Our results highlight the role of sucralose-gut microbiome interaction in regulating host health-related processes, particularly chronic inflammation.

## Introduction

Artificial sweeteners are commonly used food additives that have much higher sweetness intensities than table sugars (Gardner et al., 2012). Consumption of artificial sweeteners is increasing in the United States, and in 2008, the prevalence of consumption of beverages containing artificial sweeteners was 24.1% among adults. Sucralose, which is 600 times sweeter than sucrose, is one of the most commonly used artificial sweeteners in the market due to its extremely sugar-like taste, lack of a bitter aftertaste, stability at high temperatures, and long shelf-life (Grice and Goldsmith, 2000; Sylvetsky et al., 2012). The health effects of sucralose have been highly debated over the years. A number of previous studies concluded that sucralose is safe for its intended use as an artificial sweetener and that the body acquires no calories from sucralose (Grotz and Munro, 2009; Sylvetsky et al., 2012). Most ingested sucralose is not absorbed or metabolized and moves through the gastrointestinal tract unchanged (Roberts et al., 2000). However, this does not prove that sucralose has no effect on the gut microbiota. One study showed that a product containing sucralose altered the rat gut microbiota and induce inflammatory lymphocyte infiltration (Abou-Donia et al., 2008), but the study was considered to be deficient in several aspects (Brusick et al., 2009), including the use of high doses and a sucralose mixture instead of pure sucralose. Another study that focused on the metabolic effects of sucralose on environmental bacteria showed that sucralose can inhibit the growth of certain bacterial species (Omran et al., 2013). Therefore, sucralose may inhibit intestinal bacteria and alter the gut microbiota, and these alterations could affect host health.

The mucosal surfaces of the human intestines are host to more than 100 trillion microbes (including bacteria, fungi, viruses, and parasites) from more than 1,000 species (Ley et al., 2006; Qin et al., 2010). Gut microbes interact with the host mucosa directly via the recognition of pathogen-associated molecular patterns (PAMPs), such as lipopolysaccharide, flagellin, and bacterial DNA and RNA, by mucosal pattern recognition receptors (PRRs; Maloy and Powrie, 2011). Alternatively, the interaction can occur indirectly through secreted metabolites (Nicholson et al., 2012). These interactions are involved in maintaining symbiotic homeostasis. Increasing evidence indicates that this homeostasis is vital for human health (Holmes et al., 2011; Tremaroli and Backhed, 2012). Gut microbes can help maintain good host health by participating in digestion and fermentation of food, development of immune cells, regulation of the enteric nervous system, and prevention of colonization by pathogens (Holmes et al., 2011). The host, in turn, provides a habitat and nutrients and secretes antibodies to inhibit the aggressive expansion of microbes (Kamada et al., 2013). Being highly diverse, the gut microbiota can be shaped by various factors, including aging, diet, drugs, antibiotics, diseases, stress, exercise, and environmental pollutants (Ley et al., 2006; Nicholson et al., 2012), and if homeostasis is disrupted as a result of this shaping, many adverse outcomes may occur, such as cardiovascular disease, obesity, diabetes, allergies, and cancer (Ley et al., 2006). For example, an increased ratio of Firmicutes to Bacteroidetes was found in obese mice compared with their lean littermates (Turnbaugh et al., 2006), and obesity-related phenotypes were found to be transmissible in a study in which fecal microbes from obese and lean human twins were transferred to germ-free mice (Ridaura et al., 2013). Likewise, a remarkable increase in taurocholic acid and *Bilophila wadsworthia* induced by dietary fat promotes colitis in IL10-deficient mice (Devkota et al., 2012). Metabolites produced from dietary choline by gut microbes have been shown to be modulated in obesity, diabetes, and cardiovascular diseases (Kim et al., 2010; Wang et al., 2011).

Inflammation is one of the most common physical conditions associated with gut microbiota dysbiosis. For example, acute or chronic inflammation is the primary characteristic of inflammatory bowel diseases (IBDs; Xavier and Podolsky, 2007), of which a disrupted gut microbiota is one of the major triggers in addition to genetic factors and the host immune system, although the precise etiology remains unclear (Hill and Artis, 2010). Moreover, increasing evidence demonstrates that low-grade chronic inflammation induced by gut microbiota disruption is associated with metabolic diseases (Holmes et al., 2011). Obesity and diabetes are associated with low-grade inflammation not only in adipose tissues but also systemically. A study of bariatric surgery, a method of reducing body weight for obese individuals, showed that one gut microbe species, *Faecalibacterium prausnitzii*, is directly related to the reduction of low-grade inflammation in obesity and diabetes (Furet et al., 2010). Dyslipidemia induced by a high-fat diet results in increased levels of lipopolysaccharide (LPS), which is a pro-inflammatory mediator (Holmes et al., 2011). Moreover, chronic inflammation induced by gut microbes can drive the progression of colorectal cancer from adenoma to invasive carcinoma (Uronis et al., 2009). Thus, inflammation can be triggered and modulated by an altered gut microbiota, and exposure to compounds that can alter the gut microbiota may induce inflammation in the host.

In this study, we first used 16S rRNA gene sequencing to examine the effects of sucralose on the gut microbiome of C57BL6/J mice over a 6-month administration period. Next, we used metabolomics to profile fecal metabolome changes associated with a perturbed gut microbiome. Finally, we assessed several markers of inflammation to define the effects of sucralose consumption on host tissues. Our results show that sucralose altered the gut microbiome and associated metabolic profiles, which may contribute to inflammatory response in the mouse liver.

## Materials and methods

### Animals and sucralose exposure

Male C57BL/6J mice (~8 weeks old) purchased from the Jackson Laboratory (Bar Harbor, ME) were used in this study. Twenty male mice were housed in the University of Georgia animal facility for a week before the study and then assigned to the control or treatment group (ten mice in each group), which received tap water or sucralose (Sigma-Aldrich, MO) in tap water, respectively, for 6 months. The concentration of sucralose was 0.1 mg/ml, which was equivalent to the FDA-approved acceptable daily intake (ADI) in humans (5 mg/kg/day). Fresh solutions were made every week, and the consumption of water was measured for both groups. Standard pelleted rodent diet and tap water were provided to the mice *ad libitum*, and the mice were housed in environmental conditions of 22°C, 40–70% humidity, and a 12:12 h light:dark cycle before and during the experiment. Body weight was measured before and after the treatment. Fecal pellets were collected at baseline and at three and 6 months of treatment. Mice were euthanized with carbon dioxide and necropsied after 6 months. All experiments were approved by the University of Georgia Institutional Animal Care and Use Committee. The animals were treated humanely and with regard for alleviation of suffering.

### 16S rRNA gene sequencing of the gut microbiota

The gut microbiota was investigated using 16S rRNA gene sequencing in fecal samples at different time points. DNA was isolated from the feces of individual mice using a PowerSoil DNA Isolation Kit (MO BIO Laboratories) according to the manufacturer's instructions, and the resultant DNA was quantified and stored at −80°C until further analysis. The V4 region in the 16S rRNA gene was targeted using the universal primers 515 (5′-GTGCCAGCMGCCGCGGTAA) and 806 (5′-GGACTACHVGGGTWTCTAAT). For each sample, 1 ng of the purified fecal DNA was used as a template for amplification and then barcoded with specific indexes. The amplified products were then normalized, pooled and sequenced by an Illumina MiSeq at the Georgia Genomics Facility. Paired-end 250 × 250 (PE250, v2 kit) reads were generated at a depth of at least 25,000 reads per sample. Geneious 8.1.5 (Biomatters, Auckland, New Zealand) was used to process the raw fastq files, and the mate-paired files were trimmed to dispose of bases with an error probability higher than 0.01 and then merged. The data were then analyzed using quantitative insights into microbial ecology (QIIME, version 1.9.1) (Caporaso et al., 2010), and UCLUST was used to match operational taxonomic units (OTUs) with 97% sequence similarity against Greengenes database 13.8. The matched sequences were assigned at five different levels: phylum, class, order, family and genus. The raw data of controls and treated-mice have been uploaded into the MG-RAST server (http://metagenomics.anl.gov/) with the following job IDs: 317595, 317584, 317588, 317586, 317583, 317589, 317596, 317592, 317585, 317598, 317590, 317599, 317582, 317594, 317591, 317581, 317597, 317580, 317593, and 317587.

### Functional gene enrichment analysis

An open-source R package, Tax4Fun, was first used to analyze the enrichment of functional genes of the microbiome of each group (Asshauer et al., 2015). The output from QIIME with a SILVA database extension (SILVA 119) was used for this analysis. Tax4Fun can survey the functional genes of bacterial communities based on the 16S rRNA sequencing data and provide a good approximation to the gene profiles obtained from metagenomic shotgun sequencing methods. The results from Tax4Fun were further analyzed using Statistical Analysis of Metagenomic Profiles (STAMP) (version 2.1.3) (Parks et al., 2014).

### Fecal metabolomics analysis

Metabolites in fecal samples collected at 6 months were extracted using methanol and water as previously described (Lu et al., 2014). In brief, 20 mg of feces was disrupted in 1 ml of a methanol/water solution (1:1) with a TissueLyser at 50 Hz for 5 min, followed by centrifugation at 12,000 rpm for 10 min. The resultant upper phase was collected and dried using a SpeedVac, and the dried samples were re-suspended in 20% acetonitrile for MS analysis. Metabolomic profiling was conducted using a quadrupole-time-of-flight (Q-TOF) 6520 mass spectrometer (Agilent Technologies, Santa Clara, CA) with an electrospray ionization source interfaced with an Agilent 1200 HPLC system. The detailed method for metabolomics was published previously (Lu et al., 2012). The Q-TOF was calibrated with standard tuning solution (Agilent Technologies) daily to ensure a mass accuracy within 5 ppm. A YMC Hydrosphere C18 column was used to separate the metabolites, and all detectable molecular features in a mass range of 30–2,000 m/z were captured in the positive mode.

### Metabolomic data processing and metabolite identification

The data obtained from the HPLC-Q-TOF system were processed and analyzed as previously described (Lu et al., 2012). Briefly, the raw.d data were converted to .mzdata format using MassHunter Workstation software (Agilent), and only signals with an intensity higher than 1,000 counts were included in the subsequent analysis. Peak alignment, intensity calculations, and comparisons between the control and treatment group were performed using the XCMS Online tools. Significantly changed molecular features were profiled and searched against the Human Metabolome Database (HMDB) (http://www.hmdb.ca) and METLIN (http://metlin.scripps.edu) with a 10-ppm mass accuracy threshold. The matched exact masses were stored and used for the generation of MS/MS data to tentatively identify the metabolites. The matched molecular features were fragmented using MS/MS in the Q-TOF 6520 mass spectrometer to obtain the product ions, and the spectra were compared with the HMDB and METLIN MS/MS database to identify significantly altered metabolites, with at least two matched fragments within a 200 ppm mass accuracy as the matching threshold.

### Quantitative real-time polymerase chain reaction (qPCR)

Liver was first segmented into left lobe, media lobe, right lobe, and caudate lobe during necropsy. Each liver segment was put into a 2 ml tube, followed by immediate addition of 1 ml of RNAlater® solution (Thermo Fisher Scientific). The tubes were stored at +4°C overnight to allow the RNAlater® solution to inhibit the RNase before they were transferred to the −80°C freezer for storage. Liver samples (right lobe) treated with RNAlater® were used to isolate RNA with an RNeasy Mini Kit (Qiagen, Valencia, CA) according to the manufacturer's instructions, and the resultant RNA was digested with a DNA-free™ DNA Removal Kit (Thermo Fisher Scientific) to remove genomic DNA contamination. RNA integrity number (RIN) for each RNA sample was measured with an Agilent Bioanalyzer. The RIN typically was >9.0, indicating no RNA degradation in the samples and processing. Then, cDNA was synthesized from 1 μg of total RNA using iScript™ Reverse Transcription Supermix for RT-qPCR (Bio-Rad Laboratories, CA), and the products were diluted to 1:5 before use in subsequent reactions. Quantitative real-time PCR was performed on a Bio-Rad CFX96 Touch™ Real-Time PCR Detection System using SsoAdvanced™ Universal SYBR® Green Supermix (Bio-Rad). The sequences of the primers used for quantitative PCR were as follows: TNF-α, 5-CCCTCACACTCAGATCATCTTCT and 5-GCTACGACGTGGGCTACAG; IL-6, 5-TAGTCCTTCCTACCCCAATTTCC and 5-TTGGTCCTTAGCCACTCCTTC; IL-1β, 5-GCAACTGTTCCTGAACTCAACT and 5-ATCTTTTGGGGTCCGTCAACT; iNOS, 5-GTTCTCAGCCCAACAATACAAGA and 5-GTGGACGGGTCGATGTCAC; MMP-2, 5-CAGGGAATGAGTACTGGGTCTATTand 5-ACTCCAGTTAAAGGCAGCATCTAC; MMP-9, 5-ATCTCTTCTAGAGACTGGGAAGGAG and 5-AGCTGATTGACTAAAGTAGCTGGA; MMP-13, 5-GTGTGGAGTTATGATGATGT and 5-TGCGATTACTCCAGATACTG; and β-actin, 5-CGTGCGTGACATCAAAGAGAA and 5-TGGATGCCACAGGATTCCAT. All results were normalized to the β-actin or GAPDH gene (endogenous control). The fold change in expression over control samples was calculated using the ΔΔCT method by CFX manager software (Bio-Rad). The qPCR conditions were 95°C for 10 min, 40 cycles of 15 s at 95°C, 30 s at 60°C, and 30 s at 72°C, and a final melting curve analysis performed by raising the temperature from 65 to 95°C in 0.5°C increments for 0.05 s each. Potential genomic DNA contamination was controlled for by DNase digestion and the inclusion of a No-RT control, and technical contamination was controlled for by the inclusion of a No-template control.

### Data analysis

The difference in individual gut bacterial components between control and sucralose-treated mice at different time points was assessed with the Mothur software. A two-tailed Welch's *t*-test (*p* < 0.05) was used to compare the difference in metabolites between the control and sucralose-treated mice. Additionally, principle component analysis (PCA) was used to examine the intrinsic clusters and outliers. Partial least squares discriminant analysis (PLS-DA) and a hierarchical clustering heat map was used to visualize metabolomic difference in different groups. A two-tailed Student's *t*-test was used to determine the statistical significance of pro-inflammatory gene expression between the controls and treated mice.

## Results

### Sucralose altered the developmental dynamics of the gut microbiome

**Figure 1:**
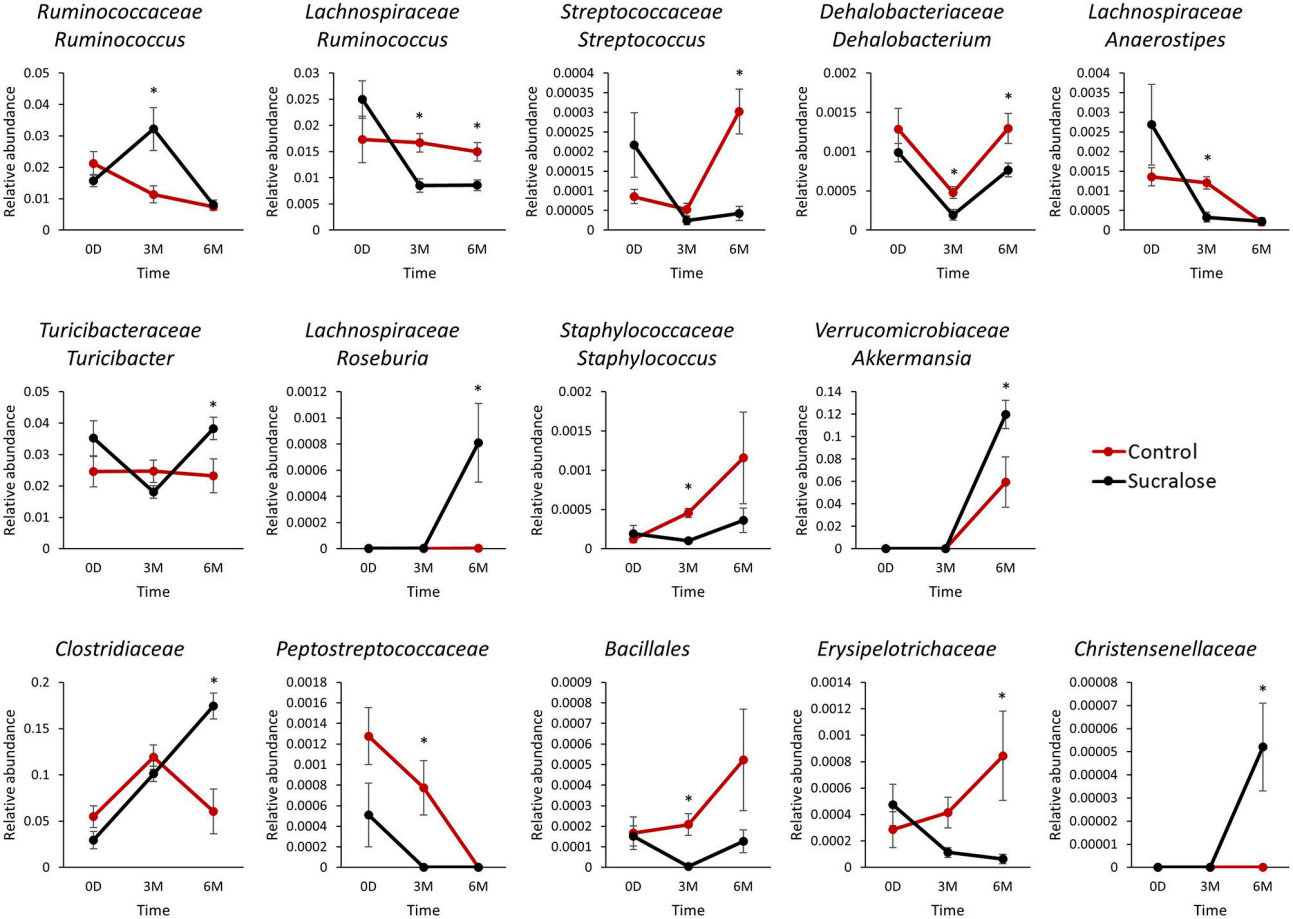
Sucralose altered the dynamics of gut microbiome development in C57BL6/J mice. Bacterial genera exhibited different patterns over time between the control and sucralose-treated mice. ^*^p < 0.05.

The gut microbiome is a dynamic system, and its bacterial composition shifts over time. Maintaining normal developmental trajectories of the gut microbiome is critical for its functions. Feces collected from both groups of mice at baseline and after three and 6 months of administration were employed to investigate the effects of sucralose on the gut microbiome. Using 16S rRNA gene sequencing, we found that 14 genera exhibited different patterns over time in sucralose-treated mice compared with control mice, as shown in Figure 1. These bacterial genera exhibited no significant difference in abundance at baseline but were significantly different after three and/or 6 months of treatment, indicating that sucralose disrupts the developmental dynamics of gut bacteria. The genera included *Turicibacteraceae Turicibacter, Lachnospiraceae Ruminococcus, Ruminococcaceae Ruminococcus, Verrucomicrobiaceae Akkermansia, Staphylococcaceae Staphylococcus, Streptococcaceae Streptococcus, Dehalobacteriaceae Dehalobacterium, Lachnospiraceae Anaerostipes, Lachnospiraceae Roseburia*, and unclassified members in Family *Clostridiaceae, Christensenellaceae, Peptostreptococcaceae, Erysipelotrichaceae* and Order *Bacillales*.

### Sucralose increased the abundance of bacterial genes related to pro-inflammatory mediators

**Figure 2:**
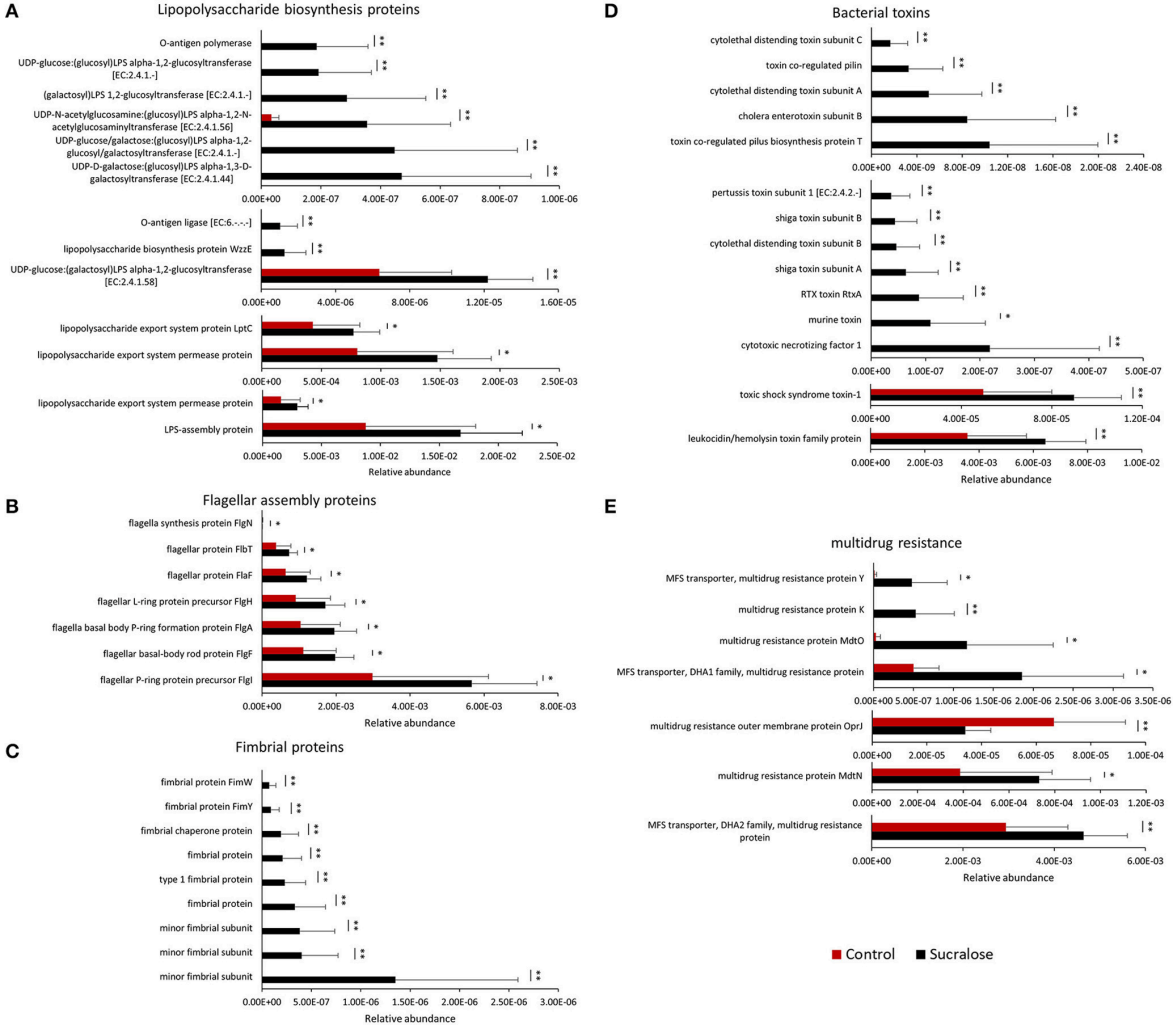
Enrichment of bacterial pro-inflammatory genes after 6 months of sucralose treatment (^*^p < 0.05, ^**^p < 0.01), including genes involved in LPS **(A)**, flagella **(B)**, and fimbriae synthesis **(C)** as well as toxins **(D)** and multidrug resistance genes **(E)**.

We next examined whether the changes in gut microbiome composition were associated with functional perturbations of the gut bacteria. Indeed, a number of bacterial functional genes were enriched in sucralose-treated mice. For example, functional gene enrichment analysis of the gut microbiome showed that genes related to bacterial pro-inflammatory mediators were highly elevated in sucralose-treated mice, as shown in Figure 2. Specifically, genes related to LPS synthesis were significantly increased after 6 months of treatment. In addition, multiple genes related to flagella protein synthesis were increased in sucralose-treated mice. Likewise, genes involved in fimbriae synthesis increased in sucralose-treated mice. Numerous bacterial toxin genes, such as toxic shock syndrome toxin-1 and shiga toxin subunits, were also elevated in sucralose-treated mice.

### Sucralose changed the fecal metabolome

**Figure 3:**
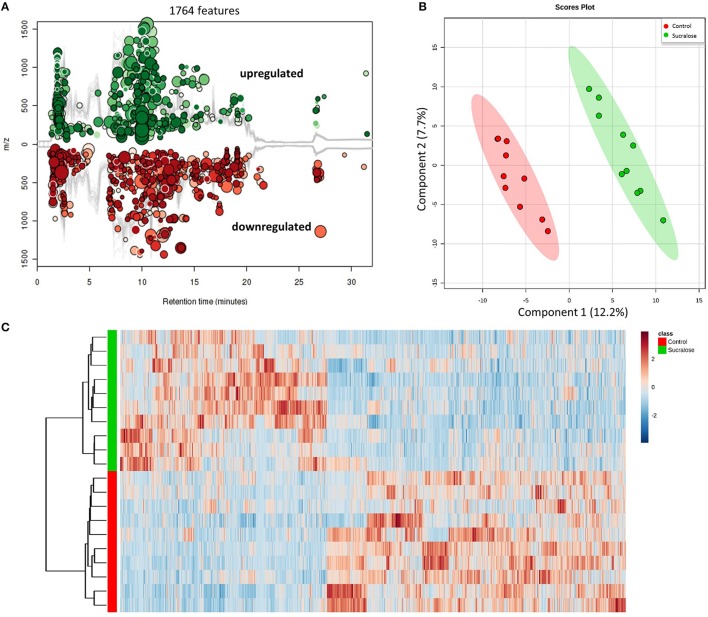
Sucralose changed the fecal metabolome, as illustrated by the Cloud plot **(A)**, PLS-DA plot **(B)**, and heat map **(C)**. A total of 1,764 molecular features were significantly different (*p* < 0.05 and fold change>1.5) between sucralose-treated mice and controls.

We next conducted metabolomic profiling to examine the functional impact of sucralose on the fecal metabolome. The combination in feces of a large quantity of gut bacteria and their metabolic products creates an ideal biological sample to assess functional changes in the gut microbiome. A total of 13,611 molecular features were detected in fecal samples, 1,764 of which were significantly different (*p* < 0.05 and fold change>1.5) between the sucralose-treated and control mice (Figure 3A), clearly indicating that sucralose perturbed the fecal metabolome. A PLS-DA plot (Figure 3B) showed a separation in the molecular patterns of the two groups, and the hierarchical clustering heat map (Figure 3C) was consistent with this result. Molecular features matched with the HMDB and METLIN database were used for metabolite identification via MS/MS. We tentatively identified 66 metabolites, including quorum sensing compounds, amino acids and derivatives, lipids, fatty acids, bile acids, and nucleic acids, among others (Supplementary Table 1).

#### Sucralose altered quorum sensing signals

**Figure 4:**
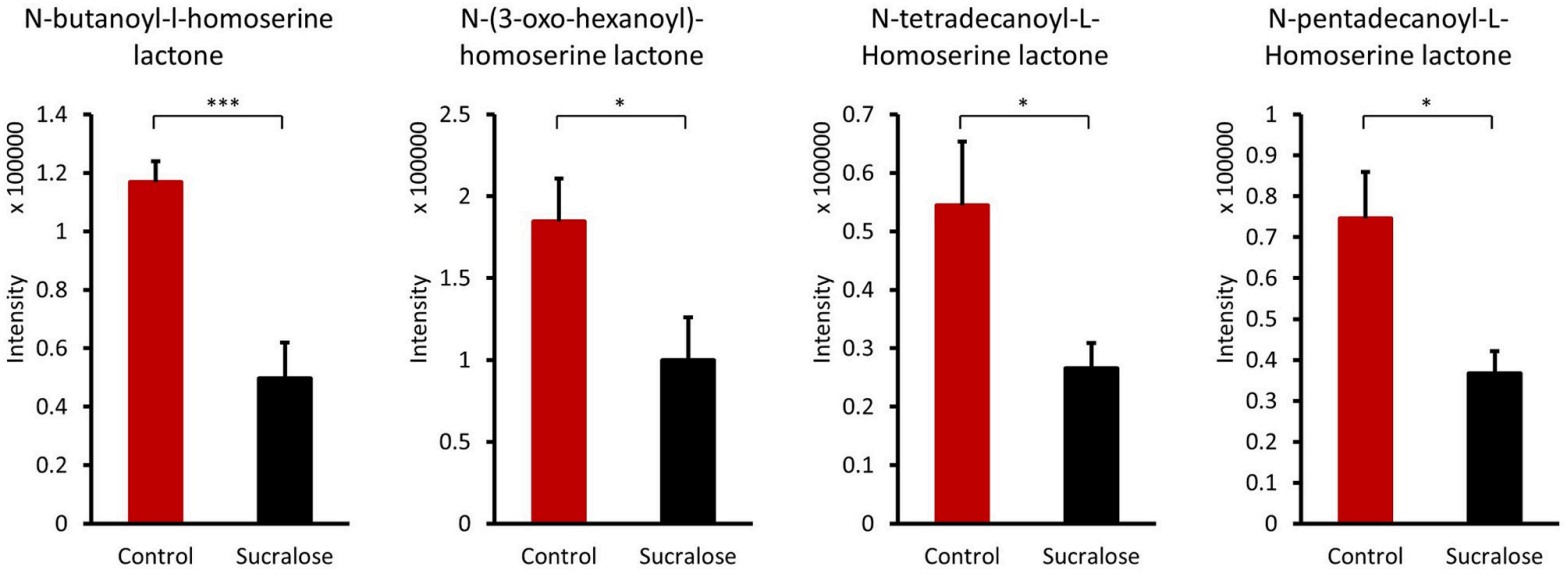
Quorum sensing signals were altered by sucralose consumption. ^*^p < 0.05, ^***^p < 0.001.

Bacteria control multicellular behaviors, such as biofilm growth and development, horizontal gene transfer, host-microbe cross-talk, and microbe-microbe interactions, by the cell-cell signaling process known as quorum sensing. Four acyl homoserine lactones known to be quorum sensing signals (Bainton et al., 1992; Winson et al., 1995; Passador et al., 1996; Stankowska et al., 2008; Lade et al., 2014) were identified: N-butanoyl-l-homoserine lactone, N-(3-oxo-hexanoyl)-homoserine lactone, N-tetradecanoyl-L-homoserine lactone, and N-pentadecanoyl-L-homoserine lactone. The reduced abundance of these quorum sensing signals in sucralose-treated mice (Figure 4) indicates that sucralose disrupts quorum sensing signaling.

#### Sucralose altered amino acids and derivatives

**Figure 5:**
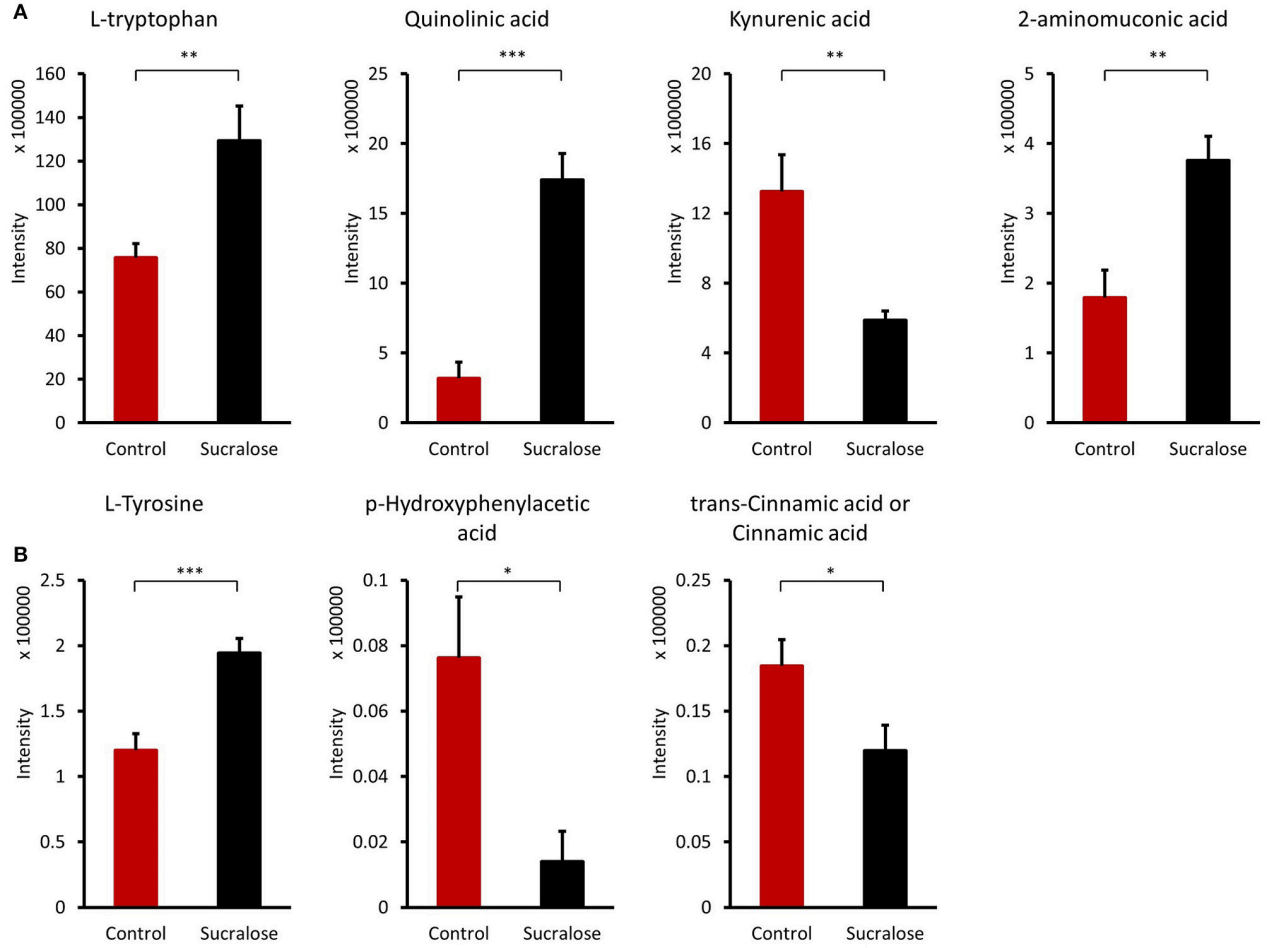
Amino acids and derivatives altered by sucralose. ^*^p < 0.05, ^**^p < 0.01, ^***^p < 0.001) **(A)** tryptophan metabolites; **(B)** tyrosine metabolites.

The gut microbiome is highly involved in the synthesis and regulation of amino acids. Amino acids, such as L-tryptophan, L-tyrosine, L-leucine, and L-isoleucine, as well as their derivatives (Table S1) were affected by sucralose treatment. Four compounds involved in tryptophan metabolism were identified, including L-tryptophan (Trp), quinolinic acid, kynurenic acid, and 2-aminomuconic acid, as shown in Figure 5A. Compared with control mice, Trp, quinolinic acid, and 2-aminomuconic acid were increased by 1.71-, 5.45-, and 2.09-fold in sucralose-treated mice, while kynurenic acid was reduced by 2.45-fold in sucralose-treated mice. For tyrosine metabolism, though L-tyrosine increased (1.62-fold), two of its metabolites, p-hydroxyphenylacetic acid and cinnamic acid, decreased by 4.63- and 1.53-fold, respectively (Figure 5B).

#### Sucralose altered bile acids

**Figure 6:**
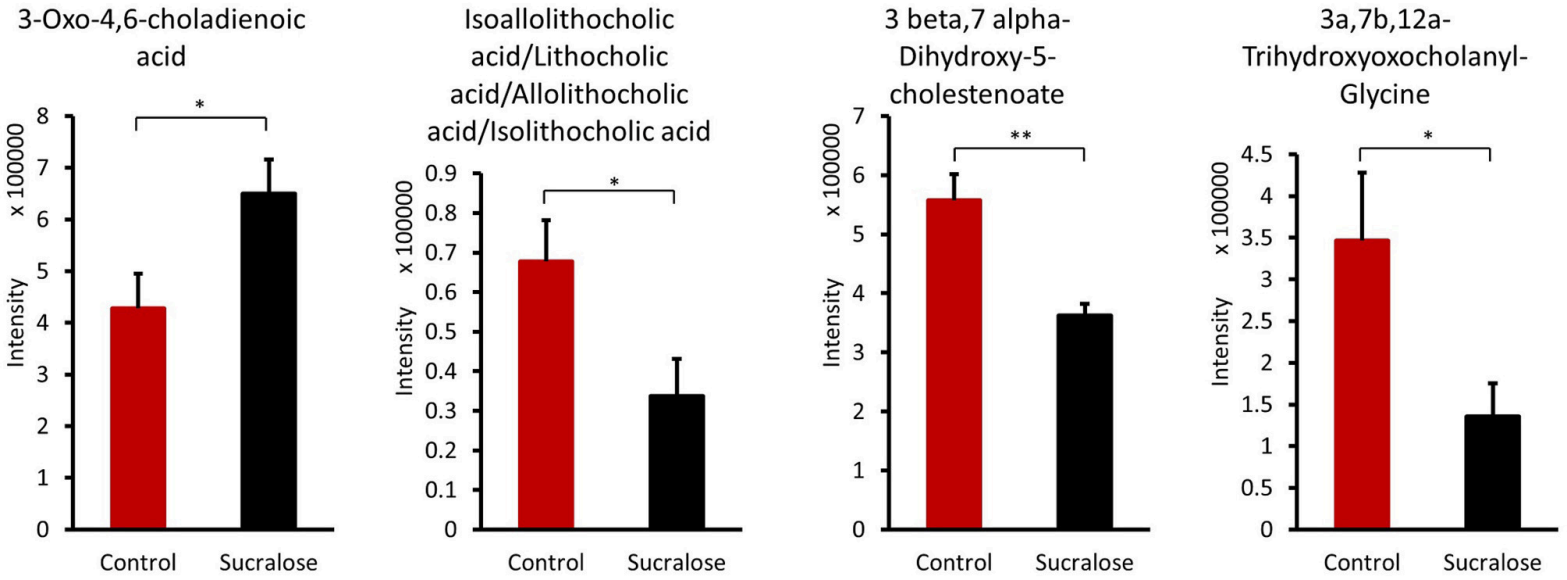
Sucralose altered bile acids in the fecal samples of mice treated with sucralose for 6 months. ^*^p < 0.05, ^**^p < 0.01.

The gut microbiome can transform primary hydrophilic bile acids into secondary hydrophilic bile acids in the large intestine through deconjugation, dehydroxylation, and dehydrogenation. Bile acids not only facilitate fat and fat-soluble vitamin absorption and maintain cholesterol homeostasis but are also viewed as signaling molecules that bind to the nuclear receptor FXR and the G-protein-coupled receptor TGR5. Several bile acids were significantly different between the control and sucralose-treated animals (Figure 6). 3-Oxo-4,6-choladienoic acid was increased in sucralose-treated mice compared with control mice, while other bile acids were reduced, including 3β,7α-dihydroxy-5-cholestenoate, 3α,7β,12α-trihydroxyoxocholanyl-glycine and lithocholic acid/isoallolithocholic acid/allolithocholic acid/isolithocholic acid.

### Sucralose induced elevated pro-inflammatory gene expression in liver

**Figure 7:**
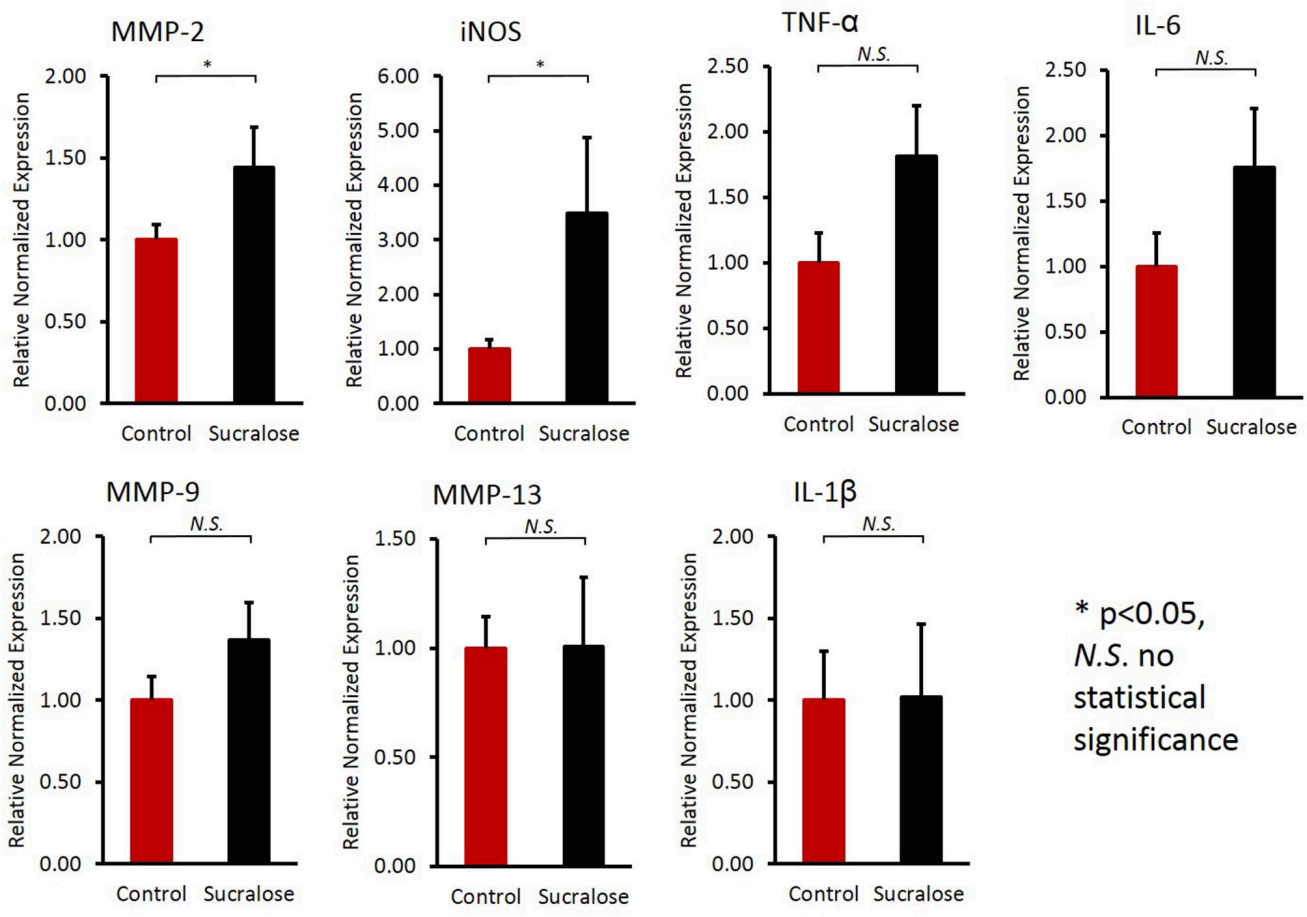
Sucralose consumption increased the gene expression of inflammatory markers in the liver, as examined by qRT-PCR. The mRNA expression of matrix metalloproteinase 2 (MMP-2) and inducible nitric-oxide synthase (iNOS) was elevated in sucralose-treated mice. ^*^p < 0.05, N.S., no statistical significance.

As described above, sucralose could increase the production of bacterial pro-inflammatory mediators, which may cause inflammatory responses in host tissues after being translocated into the host circulation. In fact, sucralose-treated mice exhibited elevated gene expression of pro-inflammatory markers in the liver (Figure 7), such as matrix metalloproteinase 2 (MMP-2) and inducible nitric-oxide synthase (iNOS).

## Discussion

The gut microbiota is a dynamic system, and maintaining a healthy balance is vital for the host (Nicholson et al., 2012). Previous studies have demonstrated that changes to the gut microbiota affect numerous host processes, such as immune system development and energy metabolism and absorption, and can also impact diseases in and beyond the GI tract (Holmes et al., 2011). Xenobiotics in the food or environment can affect the gut microbiome and host health (Lu et al., 2014; Suez et al., 2014; Gao et al., 2017). One common argument used to support sucralose safety is that the majority of sucralose is not absorbed or metabolized in the body (Grice and Goldsmith, 2000). However, we demonstrate here that sucralose can affect the gut microbiome, its metabolic functions and the host even though it passes through the GI tract unchanged.

Specifically, we investigated the effect of sucralose consumption on the gut microbiota and host in mice using 16S rRNA gene sequencing, functional gene enrichment analysis, metabolomics and real-time PCR. Sucralose consumption for 6 months altered the gut microbiome composition, fecal metabolites, and pro-inflammatory gene expression in the liver. The alterations induced by sucralose consumption could affect the development of inflammation and further influence other physiological functions in the body. This study provides a new understanding of the effect of artificial sweeteners on the gut microbiota and host health.

Sucralose has been shown to be safe using different endpoints in previous studies, but very few studies have reported its effects on the gut microbiome and, particularly, its functions (Grotz and Munro, 2009). In this study, we examined sucralose-induced gut microbiome functional perturbation, which may contribute to the development of systemic inflammation in the host. Altering the gut bacterial composition may confer an increased risk of developing inflammation in sucralose-treated mice. For example, among the 14 changed genera, several were found to be associated with host inflammation. *Ruminococcaceae Ruminococcus*, which were more abundant in sucralose-treated mice in this study, were shown to be more abundant in colonic Crohn's disease samples than in healthy samples in a previous study (Willing et al., 2010); *Streptococcaceae Streptococcus, Dehalobacteriaceae Dehalobacterium, Lachnospiraceae Anaerostipes*, and *Lachnospiraceae Ruminococcus*, which were reduced in sucralose-treated mice, were found to be negatively associated with inflammation in previous studies (Willing et al., 2010; Collins et al., 2014; Fernández et al., 2016; Munyaka et al., 2016). The functional impact of these altered gut bacteria remains to be further elucidated in the future. Nevertheless, alterations in gut microbiome composition may lead to differential functional bacterial metagenomes and metabolic capacities of the gut microbiome.

Previous studies have demonstrated that functional genes of the bacterial community are related to 16S rRNA marker genes, allowing the functional capacities of the gut microbiome to be surveyed using 16S rRNA gene sequencing (Asshauer et al., 2015). Using functional gene enrichment analysis, a number of genes related to bacterial pro-inflammatory mediators were shown to be significantly increased in the sucralose-treated gut microbiome, including genes involved in LPS synthesis, flagella protein synthesis, and fimbriae synthesis as well as bacterial toxins and drug resistance genes. LPS, flagella, and fimbriae are known PAMPs that can trigger pathological inflammation in the host, and various toxins produced by bacteria can induce toxicity in the host. LPS, a known endotoxin from the outer membrane of gram-negative bacteria, can initiate inflammatory events, such as the secretion of pro-inflammatory cytokines like interleukin-6 or tumor necrosis factor (TNF)-α (de La Serre et al., 2010). Flagella protein levels are low in a healthy gut, and high levels of flagella proteins have been shown to be associated with gut mucosal barrier breakdown and inflammation in previous studies (Cullender et al., 2013). Fimbriae play an important role in bacterial adhesion to and invasion of epithelial cells and are known virulence factors (Nakagawa, 2002). Additionally, multidrug resistance genes were increased in the sucralose-treated gut microbiome, and the increase in multidrug resistance genes and/or multidrug-resistant bacteria may lead to a more hostile gut environment (Marshall et al., 2009). These data indicate that 6 months of sucralose consumption increased the pro-inflammatory products of the gut microbiome and its ability to potentially induce systemic inflammation.

Likewise, the metabolites identified in fecal samples may be involved in regulating inflammation. For example, several amino acids were perturbed in sucralose-treated fecal metabolites in this study. In particular, we found that tryptophan metabolism was disrupted by sucralose, and this disruption was related to changes in the expression of functional genes of the gut microbiome. As shown in Supplementary Figure 1, several genes related to tryptophan metabolism were elevated, while the abundance of tryptophan and its metabolites were altered in the fecal samples. The four metabolites identified are involved in the kynurenine pathway, which is the most important tryptophan metabolism pathway, consuming 95% of the tryptophan in the body (Keszthelyi et al., 2009). The balance between two metabolites in this pathway, quinolinic acid, and kynurenic acid, plays an important role in mediating inflammation and the excitability of cells such as enteric neurons. Quinolinic acid is pro-inflammatory and excitotoxic, whereas kynurenic acid is anti-inflammatory and neuroprotective (Keszthelyi et al., 2009). Here, we found an elevated level of quinolinic acid and a decreased level of kynurenic acid. This indicates that sucralose may shift cells to a pro-inflammatory state. Likewise, tyrosine and two of its metabolites, p-hydroxyphenylacetic acid and cinnamic acid, have previously been shown to decrease the production of reactive oxygen species (ROS) in neutrophils (Beloborodova et al., 2012), and the reduced level of these compounds in our study indicated that ROS levels may be increased in sucralose-treated mice. Bacterial antioxidative enzyme genes, such as catalase and catalase-peroxidase, which respond to ROS, were also significantly enriched in sucralose-treated mice (Supplementary Figure 2). ROS can stimulate the release of pro-inflammatory cytokines (Chapple, 1997); therefore, the decrease in the two tyrosine metabolites may contribute to the development of a pro-inflammatory state. Additionally, secondary bile acids that have antimicrobial effects were decreased, which may allow the growth of pathogens (Begley et al., 2005).

Pro-inflammatory mediators, such as LPS, and metabolites can translocate into host circulation and tissues, leading to systemic inflammatory response (de La Serre et al., 2010). In accordance with this expectation, real-time PCR results showed that MMP-2 and iNOS expression was elevated in the liver of sucralose-treated mice. MMP-2 is strongly associated with inflammatory responses, because it can cleave and activate TNF-α and IL-1β, which are both pro-inflammatory cytokines that contribute to the induction of inflammation (Medina and Radomski, 2006; Wang et al., 2006). Likewise, iNOS-derived NO regulates pro-inflammatory genes and significantly contributes to inflammatory liver injury. iNOS exerts numerous effects associated with the progression of inflammatory conditions in multiple liver diseases, such as increasing the liver inflammatory response, promoting the induction of liver tumors and contributing to liver fibrosis caused by a chronic viral infection (Sass et al., 2001; La Mura et al., 2014). The expression of both MMP-2 and iNOS was found to be increased in the liver of sucralose-treated mice compared with control mice, indicating that sucralose exposure increases the risk of developing inflammation in the liver. Notably, most of the sucralose consumed passes through the GI tract unabsorbed and unchanged (Roberts et al., 2000); therefore, the inflammatory response observed in the liver was unlikely to be stimulated by sucralose directly.

**Figure 8:**
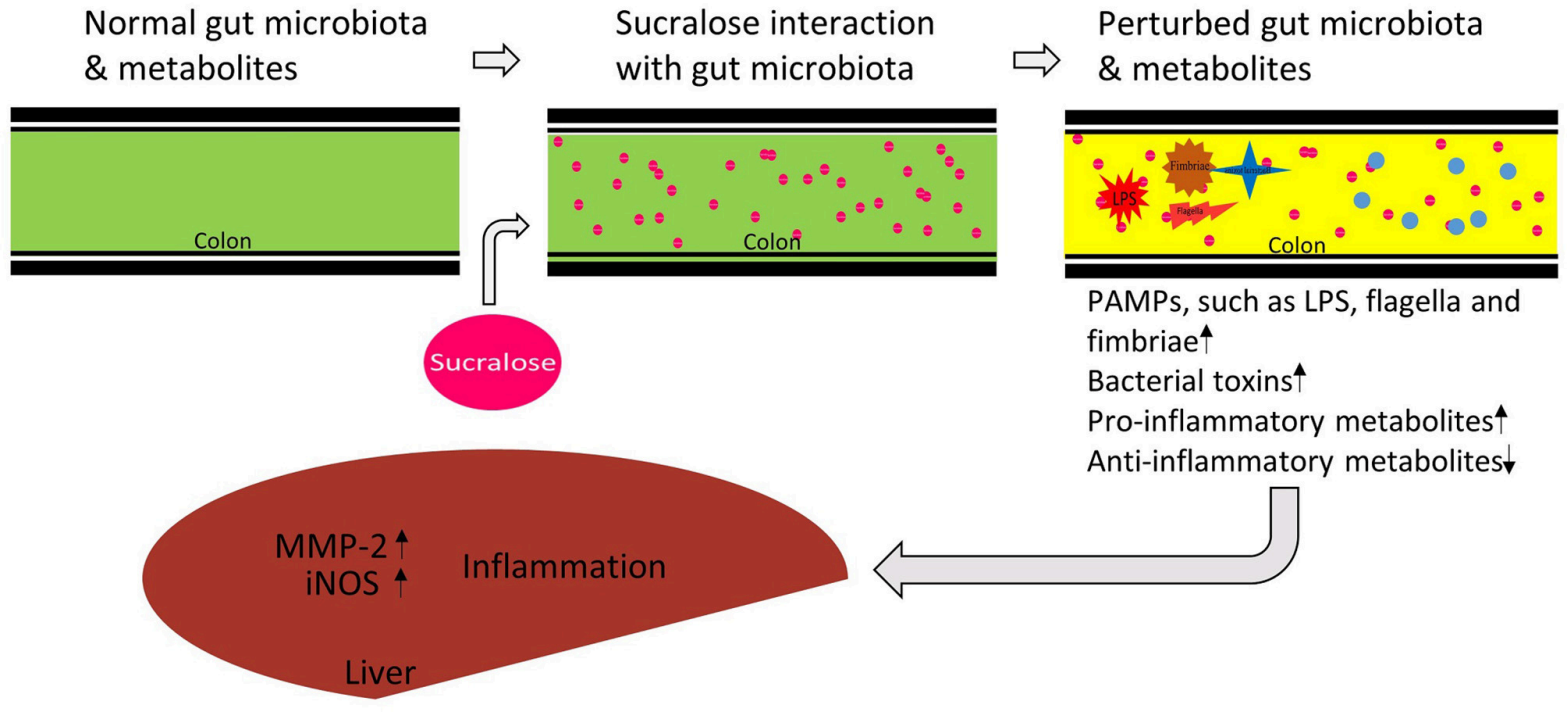
Proposed functional link between sucralose-induced gut microbiota alterations and host inflammation. Sucralose perturbs the gut microbiome and its metabolic functions, inducing the enrichment of bacterial pro-inflammatory mediators, and disrupting metabolites involved in inflammation regulation. Together, these consequences may contribute to the induction of liver inflammation in the host.

Taken together, as illustrated in Figure 8, our data show that 6-month sucralose consumption at human ADI alters the gut microbiome and its functions in mice. In particular, the enrichment of gut microbial pro-inflammatory genes and fecal metabolites suggests that sucralose alters the gut environment to release more pro-inflammatory mediators and alter functional metabolites, which may contribute to the increased expression of pro-inflammatory markers in the liver, such as iNOS and MMP-2. Notably, while the majority of ingested sucralose passes through the GI tract unabsorbed, it does disrupt the gut microbiota and its functions; therefore, our results highlight the role of sucralose-gut microbiome interaction in regulating host health-related processes, such as chronic inflammation.

There are a few limitations associated with this study. First, we only assessed inflammatory response in the liver of sucralose-treated mice by RT-PCR. Examination of host response using other endpoints and methods, such as circulating LPS and histological assessment, in related samples and tissues would be needed to better characterize the effects of sucralose in the body. Second, we conducted experiments using a single dose of sucralose at the human ADI, while human intake of sucralose is typically lower than this concentration. Our ongoing study using multiple human-relevant doses aims at better understanding time- and dose-dependent effects of sucralose on the gut microbiome and host. Third, the enrichment analysis of functional bacterial genes was performed based on the 16S rRNA sequencing data. Metagenomic shotgun sequencing and/or metatranscriptomics will further shed light on sucralose-induced functional perturbation of the gut microbiome. Finally, the identification of altered metabolites was based on the matching with the metabolite database. Future validation of key metabolites of interest with authentic compounds is warranted. Likewise, a more accurate quantitative analysis of altered metabolites using stable isotope labeled standards should be conducted.

## Author contributions

XB, HR, and KL designed the study. XB, BG, LC, and PT acquired, analyzed and interpreted the data. XB, PT, HR, and KL drafted and critically revised the manuscript, approved the version to be published, and are accountable for all aspects of the work.

### Conflict of interest statement

The authors declare that the research was conducted in the absence of any commercial or financial relationships that could be construed as a potential conflict of interest.

## Abbreviations

PAMPs: pathogen-associated molecular patterns
PRRs: pattern recognition receptors
IBD: inflammatory bowel disease
LPS: lipopolysaccharide
iNOS: inducible nitric-oxide synthase
MMP-2: matrix metalloproteinase 2
ROS: reactive oxygen species
GI: gastrointestinal.
