# Supplementary material for: Gut Microbiome Response to Sucralose and Its Potential Role in Inducing Liver Inflammation in Mice
Source: Front Physiol. 2017 Jul 24;8:487. doi: 10.3389/fphys.2017.00487 (PMC5522834; doi:10.3389/fphys.2017.00487)
Supplement: Supplementary file 1 [file DataSheet1.pdf]

Supplementary Material

Gut microbiome response to sucralose and its potential role in inducing liver inflammation in mice

Xiaoming Bian, Liang Chi, Bei Gao, Pengcheng Tu, and Kun Lu*

*** Correspondence:** Kun Lu: kunlu@unc.edu

1 Supplementary Tables and Figures

1.1 Supplementary Tables

Supplementary Table 1. Significantly altered fecal metabolites in sucralose-treated mice compared to controls.

Identified metabolites	Fold Change	p-value	m/z	retention time	Matched fragment
Quinolinic acid	5.45	0.000	168.0287	8.1	66, 94
2,3-Didehydro-pimeloyl-CoA	4.01	0.021	908.1711	11.1	136, 890
3-(3-Ethyloxiranyl)-2-propenal	3.32	0.025	149.0582	10.9	77, 91, 149
N-Acetylhistidine	3.06	0.000	236.0416	2.1	54, 60, 108, 110, 134, 180.
5,8,11,14-all-cis-tetracosanoylethanolamide/17,17-dimethyl-5,8,11,14-all-cis-docosatetraenoylethanolamide/(16,16-dimethyldocosa-cis-5,8,11,14-tetraenoyl) ethanolamine	2.82	0.018	426.3371	17.8	44, 62
2-Oxosuccinamate	2.66	0.000	169.9847	2.0	58, 70, 96, 97
Ne,Ne dimethyllysine	2.58	0.024	213.0984	1.5	84, 102, 110, 129, 139
L-gamma-glutamyl-L-isoleucine	2.21	0.007	261.1453	8.5	56, 69, 84, 86, 102, 130, 198, 226
L-gamma-glutamyl-L-leucine	2.21	0.007	261.1453	8.5	56, 69, 84, 86, 102, 130, 198, 226
PG(18:4(6Z,9Z,12Z,15Z)/0:0)	2.15	0.047	505.2557	4.0	213, 231, 487
2-aminomuconic acid	2.09	0.001	158.0456	2.8	68, 85, 95, 98, 114, 122, 158
L-prolyl-L-proline	2.01	0.034	213.125	7.9	70, 98, 195

(all-Z)-7,10,13-Docosatrienoic acid	1.99	0.008	357.279	15.2	41, 55, 69, 83, 95, 135, 145, 147, 161, 175, 201, 215, 203, 233, 247, 275, 289, 339, 357
L-Isoleucine/L-leucine	1.94	0.001	132.1015	31.5	44, 86
Citrulline	1.87	0.006	214.0568	2.1	43, 70, 86, 151
N-oleoyl leucine	1.87	0.012	396.3509	19.1	59, 69, 86, 125, 239, 247, 350, 396
N-oleoyl isoleucine	1.87	0.012	396.3509	19.1	59, 69, 86, 125, 239, 247, 350, 396
N6-Acetyl-L-lysine	1.76	0.008	189.1232	4.1	56, 84, 130
LysoPE(0:0/14:0)	1.75	0.036	426.2635	17.6	44, 62, 198, 285, 365
L-Tryptophan	1.71	0.009	205.0972	8.2	74, 91, 115, 118, 127, 130, 142, 143, 144, 146, 159, 170, 188
L-Tyrosine	1.62	0.000	182.0818	11.5	77, 91, 118, 123, 136
3-Oxo-4,6-choladienoic acid	1.52	0.030	371.256	12.4	55, 81, 107, 121, 133, 159, 173, 257, 269, 327, 353, 355, 371
Cinnamic acid	-1.53	0.032	149.0605	10.2	77, 103
3 beta,7 alpha-Dihydroxy-5-cholestenoate	-1.54	0.002	455.3111	11.6	55, 123, 129, 131, 269, 313, 363, 375, 391, 409, 419, 437, 455
Acetylagmatine	-1.57	0.041	195.1217	2.2	41, 43, 55, 70, 82, 87, 109, 111, 122
Adenosine	-1.6	0.036	268.1037	4.4	57, 136
Palmitaldehyde	-1.6	0.006	263.237	18.1	43, 55, 69, 93, 119, 121, 127, 163, 245
Palmitelaidic acid	-1.67	0.021	255.2317	17.5	43, 55, 57, 69, 81, 83, 109, 137, 219
Pyridoxal	-1.72	0.005	168.0648	8.3	67, 77, 79, 94, 106, 122
C17 Sphinganine	-1.83	0.040	288.2896	13.7	43, 57, 60, 71, 85, 99, 113, 155, 209, 240, 270
N-(3-oxo-hexanoyl)-homoserine lactone	-1.85	0.034	236.0897	4.7	43, 56, 84, 95, 102, 164, 178, 190, 236

3-Dehydrosphinganine	-1.86	0.047	300.2902	14.0	41, 43, 55, 57, 60, 71, 85, 99, 127, 239, 264, 282, 300
N,N-Dimethylsphingosine	-1.9	0.015	328.321	15.2	44, 55, 57, 58, 67, 69, 81, 83, 95, 109, 280
Adenine	-1.97	0.025	136.0618	4.6	55, 65, 82, 92, 94, 109, 119, 136
Lithocholic acid /isoallolithocholic acid /allolithocholic acid/ isolithocholic acid	-2	0.027	399.2907	11.3	111, 123, 187, 193, 201, 255, 257, 275, 281, 283, 301, 313, 319, 333, 351, 363, 365, 381, 399
Guanine	-2.02	0.002	152.0565	6.8	53, 57, 85, 158
N-pentadecanoyl-L-Homoserine lactone	-2.03	0.010	364.226	18.2	57, 83, 85, 109, 308
Nonadeca-10(Z)-enoic acid	-2.04	0.036	297.278	31.2	41, 55, 57, 71, 85
N-tetradecanoyl-L-Homoserine lactone	-2.05	0.035	350.2123	17.7	57, 83, 84, 85, 123
L-Dopa	-2.05	0.000	198.0762	3.0	65, 77, 79, 107, 109, 135, 152
L-2,3-Dihydrodipicolinate	-2.11	0.023	208.0023	2.2	118, 136, 144, 164, 208
2-Furoylglycine	-2.11	0.023	208.0023	2.2	39, 95
3-Dehydroquinate	-2.17	0.006	191.0539	12.0	43, 117, 145, 173
N(6)-Methyllysine	-2.18	0.033	161.127	1.5	44, 58, 70, 84, 98, 115
Kynurenic acid	-2.25	0.006	190.0495	12.0	89, 116, 144
N-butanoyl-l-homoserine lactone	-2.35	0.000	172.0977	9.5	43, 56, 70, 84, 154
3a,7b,12a-Trihydroxyoxocholanyl-Glycine	-2.55	0.036	466.3139	12.6	69, 85, 448, 466
Hydroxychlorobactene glucoside	-2.61	0.007	735.4571	11.2	237, 423, 499, 717
Sterol	-2.92	0.016	271.2057	14.1	43, 187, 253, 243, 271
N,N-dimethyl arachidonoyl amine	-2.95	0.038	332.2957	21.3	57, 105
DAT(16:0/23:0(2Me[S],3OH[S],4Me[S],6Me[S]))	-3.02	0.009	997.7105	10.3	163, 221, 795, 961
PC(P-17:0/0:0)	-3.42	0.041	494.3631	21.3	311, 494
LysoPC(14:1(9Z))	-3.51	0.000	466.291	11.0	222, 258, 283, 448, 466
PA(12:0/18:0)/PA(15:0/15:0)	-3.96	0.019	659.4018	12.3	81, 339, 561
N-linoleoyl taurine	-4.09	0.038	426.2039	26.9	57, 111, 126

PA(13:0/20:5(5Z,8Z,11Z,14Z,17Z))/PA(20:5(5Z,8Z,11Z,14Z,17Z)/13:0)	-4.45	0.005	675.4003	11.8	577, 657
p-Hydroxyphenylacetic acid	-4.63	0.010	153.055	11.0	57, 67, 77, 107
(20R/S)-24-Hydroxygeminivitamin D4	-9.27	0.001	519.4065	12.5	139, 427, 443, 483, 501, 519

1.2 Supplementary Figures

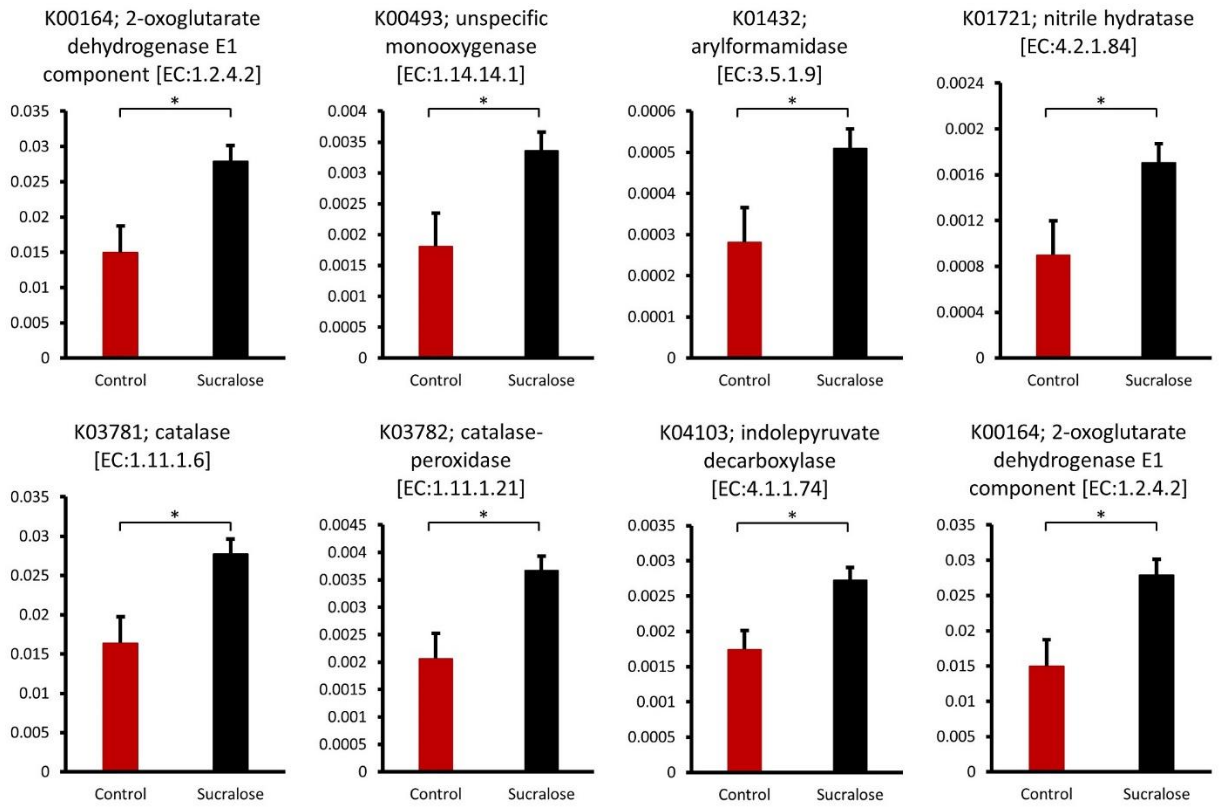

Supplementary Figure 1. Enrichment of bacterial genes related to tryptophan metabolism in the gut microbiome of sucralose-treated mice (*p<0.05).

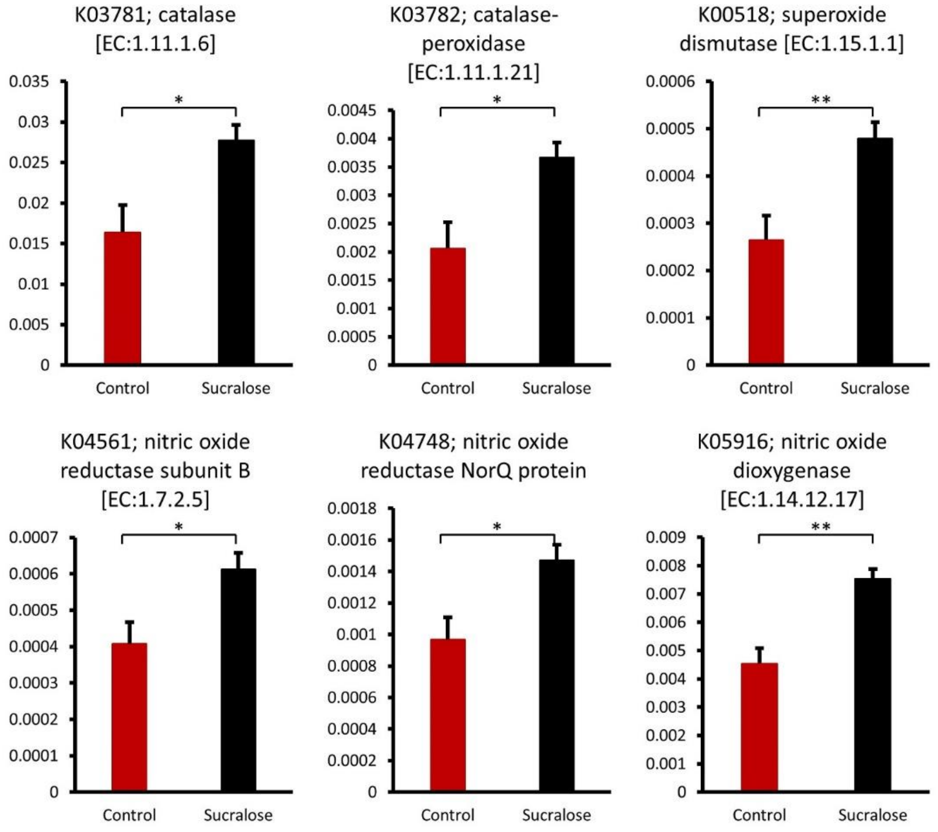

Supplementary Figure 2. Increase of bacterial genes of antioxidative enzymes in the gut microbiome of sucralose-treated mice (*p<0.05, **p<0.01)
